# Individualizing Risk of Multidrug-Resistant Pathogens in Community-Onset Pneumonia

**DOI:** 10.1371/journal.pone.0119528

**Published:** 2015-04-10

**Authors:** Marco Falcone, Alessandro Russo, Maddalena Giannella, Roberto Cangemi, Maria Gabriella Scarpellini, Giuliano Bertazzoni, José Martínez Alarcón, Gloria Taliani, Paolo Palange, Alessio Farcomeni, Annarita Vestri, Emilio Bouza, Francesco Violi, Mario Venditti

**Affiliations:** 1 Department of Public Health and Infectious Diseases, Policlinico Umberto I, “Sapienza” University of Rome, Rome, Italy; 2 Department of Emergency Medicine, Policlinico Umberto I, “Sapienza” University of Rome, Rome, Italy; 3 Unit of Infectious Diseases, Department of Medical and Surgical Sciences, St. Orsola-Malpighi Hospital, Alma Mater Studiorum University of Bologna, Bologna, Italy; 4 Department of Internal Medicine and Medical Specialties, Policlinico Umberto I, “Sapienza” University of Rome, Rome, Italy; 5 Department of Microbiology, General Hospital, Ciudad Real, Spain; 6 Department of Clinical Medicine, Policlinico Umberto I, “Sapienza” University of Rome, Rome, Italy; 7 Department of Public Health and Infectious Diseases-Statistics Section, Policlinico Umberto I, “Sapienza” University of Rome, Rome, Italy; 8 Department of Clinical Microbiology and Infectious Diseases, Hospital General Universitario Gregorio Marañón, Madrid, Spain; D'or Institute of Research and Education, BRAZIL

## Abstract

**Introduction:**

The diffusion of multidrug-resistant (MDR) bacteria has created the need to identify risk factors for acquiring resistant pathogens in patients living in the community.

**Objective:**

To analyze clinical features of patients with community-onset pneumonia due to MDR pathogens, to evaluate performance of existing scoring tools and to develop a bedside risk score for an early identification of these patients in the Emergency Department.

**Patients and Methods:**

This was an open, observational, prospective study of consecutive patients with pneumonia, coming from the community, from January 2011 to January 2013. The new score was validated on an external cohort of 929 patients with pneumonia admitted in internal medicine departments participating at a multicenter prospective study in Spain.

**Results:**

A total of 900 patients were included in the study. The final logistic regression model consisted of four variables: 1) one risk factor for HCAP, 2) bilateral pulmonary infiltration, 3) the presence of pleural effusion, and 4) the severity of respiratory impairment calculated by use of PaO2/FiO2 ratio. A new risk score, the ARUC score, was developed; compared to Aliberti, Shorr, and Shindo scores, this point score system has a good discrimination performance (AUC 0.76, 95% CI 0.71-0.82) and calibration (Hosmer-Lemeshow, χ2 = 7.64; p = 0.469). The new score outperformed HCAP definition in predicting etiology due to MDR organism. The performance of this bedside score was confirmed in the validation cohort (AUC 0.68, 95% CI 0.60-0.77).

**Conclusion:**

Physicians working in ED should adopt simple risk scores, like ARUC score, to select the most appropriate antibiotic regimens. This individualized approach may help clinicians to identify those patients who need an empirical broad-spectrum antibiotic therapy.

## Introduction

Pneumonia caused by multidrug-resistant (MDR) pathogens traditionally has been confined to the hospital setting, but the emergence of MDR bacteria that cause pneumonia in the community has created the need to identify risk factors for acquiring resistant pathogens by evaluating the contacts patients have with the healthcare environment [1–2]. Prior hospitalization, residency in a nursing home, attendance of hemodialysis centers, and receipt of domiciliary care are some of the risk factors for acquiring the resistant pathogens included in the healthcare-associated pneumonia (HCAP) definition [3,4,5,6]. Some studies have shown that the current definition of HCAP is no longer sufficient for early identification of patients with an increased risk of pneumonia due to MDR bacteria [1,5,7], and more recently three studies [8,9,10] have proposed new scores to discriminate patients with an increased risk of developing community-onset pneumonia due to MDR pathogens.

Considering the aforementioned, the aim of our study is a prospective evaluation of patients with community-onset pneumonia due to MDR pathogens, to compare this population to patients without a MDR etiology, and to define risk factors and outcomes of patients acquiring MDR bacteria. Furthermore, the aim is to evaluate the performance of existing scores [8,9,10] and to derive and to validate a new bedside risk score for an early identification of patients with community-onset pneumonia due to MDR pathogens in the Emergency Department (ED).

## Patients and Methods

### Study Design and Study Patients

This was an open, observational, prospective study of consecutive patients coming from the community who were admitted, from January 2011 to January 2013, in the Policlinico Umberto I of Rome, a teaching 1100-bed Hospital. The Institutional Review Board of the Policlinico Hospital approved the study. Patients >18 years of age who satisfied the criteria for pneumonia were included in the study. The patient enrollment process was: consecutive patients coming from the community and admitted to our hospital from the ED were initially screened. Two physicians (MF and AR) performed a screening twice per day in the ED looking for patients with a suspicion of pneumonia. Once patients were identified they signed an informed consent and then were followed during hospitalization until discharge or death. Patients taking immunosuppressive therapy, patients with hospital-acquired pneumonia (HAP), or discharged or dead within ≤48 hours were excluded from the final analysis.

Data on previous antibiotic therapy as well as other risk factors for MDR organisms were derived from the following sources: a) history taken from patients and/or relatives; b) discharge letters and summaries if patients were previously hospitalized in other facilities, and c) electronic charts if patients were previously hospitalized or seen in the clinics at the Policlinico Umberto I of Rome, Italy.

The following data were recorded: demographics; past medical history; severity of symptoms on admission; pneumonia severity index (PSI) and CURB-65 score (confusion, urea nitrogen, respiratory rate, blood pressure, >65 years of age) [11]; clinical, laboratory, and radiological findings on admission; microbiological data; empiric antibiotic therapy; hospital stay; in-hospital mortality (for a total of 133 variables).

### Study Definitions

Pneumonia was defined, according to the standard definitions of the Centers for Disease Control and Prevention (CDC) [12]. Severe sepsis was defined as sepsis with sepsis-induced organ dysfunction or tissue hypoperfusion (manifesting as hypotension, elevated lactate, or decreased urine output); septic shock as severe sepsis plus persistently low blood pressure following the administration of intravenous fluids [13]. Pleural effusion was detected using by chest X-ray. Length of hospital stay (LOS) was calculated as the number of days from the date of admission to the date of discharge or death. We classified patients as having HCAP if they had attended a hospital or hemodialysis clinic or received intravenous chemotherapy in the past 30 days, had been admitted to an acute-care hospital for at least 2 days or had surgery in the past 90 days, or resided in a nursing home or long-term care facility. We classified patients as having hospital-acquired pneumonia (HAP) if they received their diagnosis after being hospitalized for more than 72 hours or within 10 days of leaving the hospital.

### Microbiological Analysis and Empiric Antibiotic Therapy

Microbiological examinations performed on sputum, urine, and blood during the first 24 hours after admission and according to standards of practice were evaluated for assessment of microbial etiology. Results of pleural puncture, tracheobronchial aspirates, and bronchoalveolar lavage fluid, when performed, were also considered as well as serologic tests for *Chlamydophila pneumoniae*, *L*. *pneumophila*, or *Mycoplasma pneumoniae*. Clinical isolates obtained from patients were identified in the Microbiology Laboratory of our hospital using the automatic system VITEK 2 (bioMérieux Inc., Hazelwood, MO) [14]. Antibiotic resistance profiles, including the MIC for each isolate, were assessed using the abovementioned automatic system. Microbiological results were reviewed, and a microbiological cause was assigned independently by 2 of the investigators (MF and AR). The etiology was considered definite if 1 of the following criteria was met: positive blood culture in the absence of an apparent extrapulmonary focus; positive bacterial culture of pleural fluid; positive urinary antigen for *Legionella pneumophila* (Binax Now, Trinity Biotech); positive urinary antigen for *Streptococcus pneumoniae* (Binax Now, Emergo Europe); a bacterial yield in cultures of valid sputum (>25 polymorphonuclear cells and <10 epithelial cells per power field) of ≥10^6^ colony forming units (CFU)/mL, tracheobronchial aspirates of ≥10^5^ CFU/mL, bronchoalveolar lavage fluid of ≥10^4^ CFU/mL, and protected specimen brush cultures of ≥10^3^ CFU/mL; and occurrence of seroconversion (a 4-fold rise in immunoglobulin G [IgG] titers for *Chlamydophila pneumoniae* [1:512] or a rise in immunoglobulin M [IgM] titers for *C*. *pneumoniae* [1:32] and *Mycoplasma pneumoniae* [any titer]). When ≥ 2 microbiological causes were present, the patient was considered to have a polymicrobial infection. Patients for whom no microbiological tests were performed and patients with negative microbiological results were considered to have disease of an unknown etiology.

A patient was considered to have a MDR pathogens if one of the following was isolated: methicillin-resistant *Staphylococcus aureus* (MRSA), *Stenotrophomonas maltophilia*, extended spectrum β-lactamase (ESBL)–producing or carbapenem-resistant Enterobacteriaceae. For the remaining cases MDR was defined as isolation of a bacterial strain non-susceptible to at least one agent in three or more antimicrobial categories [15].

We defined empirical antibiotic therapy as antibiotics administered on the first day of therapy for pneumonia and considered an empirical antibiotic regimen as adherent current international guidelines [16–17]. We analyzed the appropriateness of antibiotic therapy for all patients with an etiologic diagnosis according to susceptibility test criteria for lower respiratory tract pathogens. We rated an antimicrobial treatment as inadequate if 1 or more of the organisms present were known to have intrinsic resistance or were found to be resistant through susceptibility testing.

### Study Groups and Endpoints

Two study groups were identified among the study population according to the isolation of MDR bacteria: group “MDR+”, patients with isolation of ≥ 1 resistant pathogen, and group “MDR-“, patients without MDR isolation. The microbiological endpoint was the actual isolation of an MDR pathogen.

The primary objective of this study was an evaluation of the performance of three existing scoring systems and the derivation of a new scoring tool for the identification of patients with pneumonia due to MDR organisms.

The secondary objective is an external validation of our score in a multicenter Spanish population of patients with pneumonia.

### External validation

The obtained score was validated on an external cohort of adult patients with pneumonia hospitalized in 59 IMDs of 53 Spanish hospitals participating at a prospective observational multicenter study, carried-out over a 2-week period, first week in February 2013 and second week in June 2013 (Estudio de Neumonia en Medicina Interna II). Study methods were reported in a previous study [18]. Briefly, all the participating IMDs reported data on all the adult patients treated for pneumonia at their departments during the study period through a web site. Collected data were systematically reviewed by the coordinating investigator (MG) before they were entered in the database.

### Statistical analysis

Continuous variables are presented as mean±SD, and differences were evaluated by t-test. Categorical variables were expressed as count and percentages and compared by chi-square test or Fisher’s exact test, as appropriate.

We performed logistic regression on MDR isolation to determine the best predictors. We selected potentially useful baseline characteristic predictor variables by minimization of Akaike Information Criterion (AIC), given that the main target for analysis is prediction. For reasons of parsimony, we then discarded non-significant variables from the model minimizing AIC. Model stability and absence of collinearity was checked by computing Variance Inflaction Factors (VIF). A VIF < 3 usually indicates no issues with the model. Candidate variables included: demographic characteristics (age, sex), ≥2 comorbidities, pleural effusion, HCAP, PaO2/FiO2 <300, bilateral pulmonary infiltration, fever > 38°C. A risk score named ARUC score (**A**ssessment of **R**isk of multidr**U**g resistant pathogens in **C**ommunity-onset pneumonia) was developed. The resulting score values were derived by rounding the beta coefficients (logarithm of the odds-ratios).

We evaluated discrimination using receiver operating characteristic curves (ROC). We compared ROC curves for the different scores; we adjust the probability using the Sidak method. The calibration of the model was evaluated by the goodness-of-fit Hosmer-Lemeshow χ2 statistic.

We calculated sensitivity, specificity, negative and positive predictive values (with 95% confidence intervals) for the cut-off point of the score in order to predict the MDR status. We also calculated negative and positive likelihood ratios (with 95% confidence intervals). All tests were two-tailed, and a P value < 0.05 was considered significant. All computations were carried out with R version 3.0.2, SPSS 20.0 for Windows (SPSS Inc., Chicago, IL) and STATA v.12.

## Results

**Fig 1:**
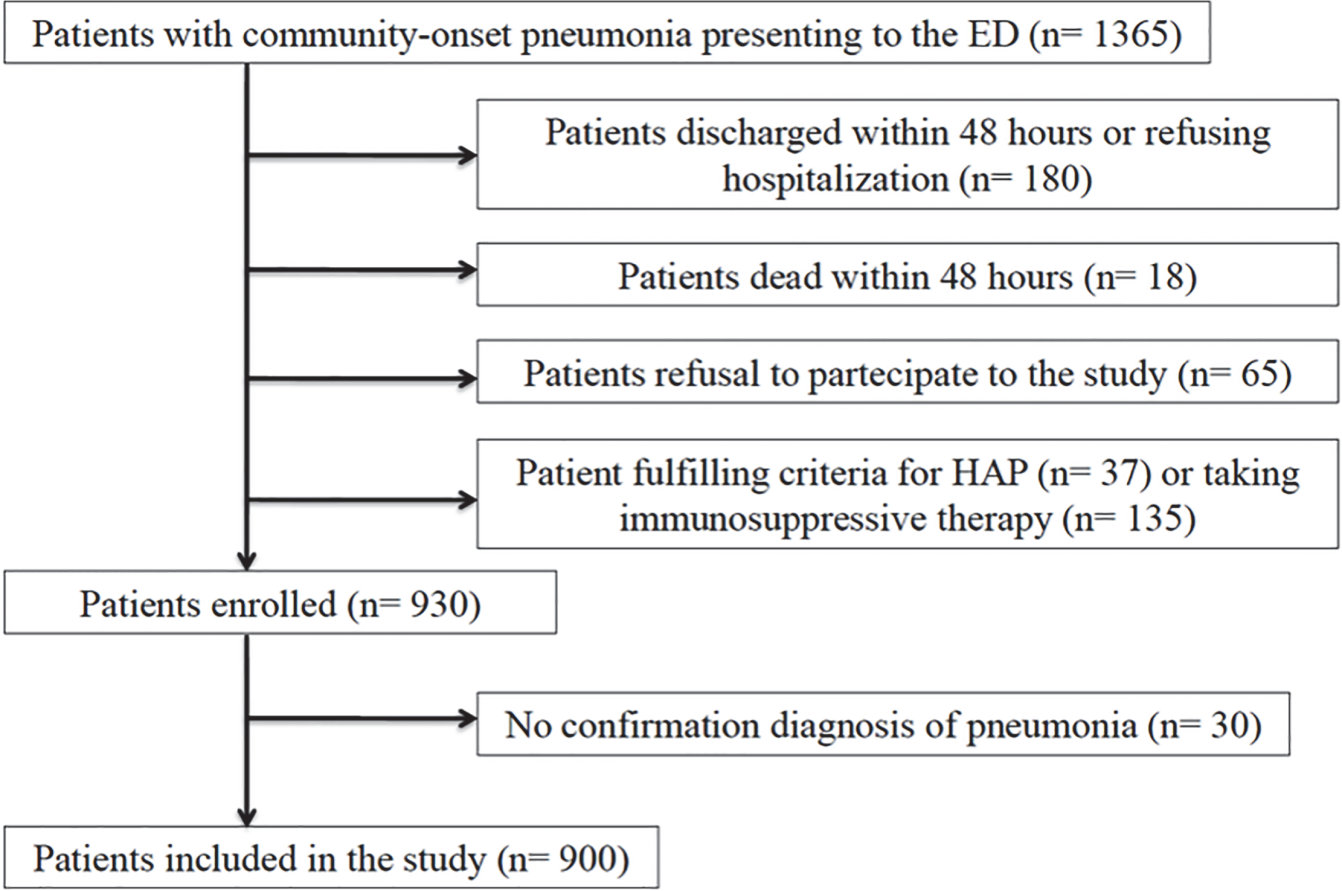
Study flow diagram. Legend. ED: emergency department; HAP: hospital-acquired pneumonia.

During the study period a total of 1,365 patients were assessed for eligibility, and 900 were finally included in the study: 536 patients had CAP and 364 HCAP. A consort diagram describing the study flow is presented in **Fig 1**.

Overall, an etiologic diagnosis was obtained in 300 patients (33.3%). Pneumonia was caused by a MDR pathogen (group MDR+) in 99 cases (11%), while the remaining patients had no MDR pathogen isolation (group MDR-). Etiologic agents of pneumonia in our study population are described in **Table 1.**

**Table 1:** Etiology of 300 isolations in the study population.

**Isolation of MDR strain n = 99 patients**		**Isolation of non-MDR strain n = 201 patients**	
MRSA	42 (42.4%)	*Streptococcus pneumoniae*	69 (34.3%)
*Pseudomonas aeruginosa*	11 (11.1%)	MSSA	31 (15.4%)
*Klebsiella pneumonia*	10 (10.1%)	*Mycoplasma pneumoniae*	15 (7.5%)
*Serratia marcescens*	8 (8.1%)	*Pseudomonas aeruginosa*	15 (7.5%)
*Escherichia coli*	7 (7.1%)	*Klebsiella pneumoniae*	14 (7%)
MRSA + *P*. *aeruginosa*	6 (6.1%)	*Haemophilus influenzae*	13 (6.6%)
*Acinetobacter baumannii*	6 (6.1%)	*Clamydia pneumoniae*	10 (4.9%)
*Enterobacter cloacae*	4 (4%)	*Mycobacterium spp*	10 (4.9%)
*E*. *cloacae + S*. *maltophilia*	2 (2%)	*Legionella pneumophila*	10 (4.9%)
MRSA + *A*. *baumannii*	2 (2%)	*Enterobacter cloacae*	8 (4%)
*Stenotrophomonas maltophilia*	1 (1%)	*Escherichia coli*	6 (3%)

Legend. MSSA: methicillin-sensitive *Staphylococcus aureus*; MRSA: methicillin-resistant *Staphylococcus aureus*; MDR: multidrug-resistant.

**Fig 2:**
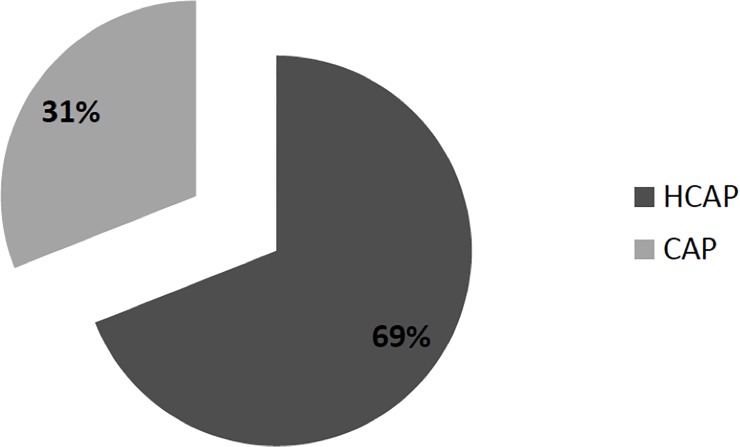
Distribution of MDR pathogens in CAP and HCAP populations. Legend. MDR: multidrug-resistant; CAP: community-acquired pneumonia; HCAP: health-care associated pneumonia.

At least one microbiological test was performed in the 70% of patients enrolled. As described in **Fig 2,** the majority of patients with MDR isolates belonged to the HCAP group (68.7%) but a significant percentage (31.3%) was also encountered in the CAP group.

Comparison of demographics, clinical features and outcome of patients included in the MDR+ and MDR-groups are summarized in **Table 2**.

**Table 2:** Univariate analysis of MDR-group compared to patients without MDR isolation.

**Variables**	**MDR+**	**MDR-**	**OR**	**95% CI**	**p**
	**n = 99 patients**	**n = 801 patients**			
Age (median)	78	82	1.02	1.00–1.03	**0.029**
Male sex	55 (61.2%)	490 (55.5%)	1.26	0.52–1.21	0.281
PSI IV-V class	98 (99%)	637 (69.7%)	2.71	1.78–4.15	**<0.001**
Adherence to guidelines	31 (26%)	421 (45.9%)	0.41	0.26–0.62	**<0.001**
≥ 2 comorbidities	37 (37.4%)	161 (20.1%)	2.27	1.28–3.34	**<0.001**
Charlson Comorbidity Index (median)	4.7	3.2	3.12	2.22–5.34	**<0.001**
Aliberti score ≥ 3	70 (70.7%)	338 (42.2%)	3.72	2.1–5.7	**<0.001**
Shorr score ≥ 1	69 (69.7%)	303 (37.8%)	4.1	2.56–6.11	**<0.001**
Shindo score ≥ 2	60 (60.6%)	299 (37.3%)	2.86	1.87–4.48	**<0.001**
Heart failure	69 (69.7%)	217 (27.1%)	4.44	2.93–6.77	**<0.001**
Chronic hepatitis	16 (16.1%)	67 (8.3%)	1.78	1.09–2.76	**0.016**
Diabetes	19 (19.2%)	151 (18.8%)	1.28	0.78–2.14	0.893
Renal failure	43 (43.4%)	144 (17.9%)	2.56	1.49–4.51	**<0.001**
COPD	40 (40.4%)	255 (31.8%)	1.76	0.91–2.22	0.09
Dementia	21 (21.2%)	180 (22.4%)	1.08	0.59–1.45	0.898
HCAP	68 (68.7%)	296 (36.9%)	4.11	2.34–6.12	**<0.001**
Neoplasm	21 (21.2%)	170 (21.2%)	0.29	0.12–0.87	1.0
Pleural effusion	56 (56.5%)	329 (41.1%)	2.72	1.79–3.84	**0.005**
Malnutrition	48 (48.5%)	101 (12.6%)	4.85	3.02–7.22	**<0.001**
PPI/H2 blockers	54 (54.5%)	239 (29.8%)	2.82	1.89–4.71	**<0.001**
Previous surgery (30 days)	13 (13.1%)	19 (2.3%)	5.9	2.81–12.1	**<0.001**
Bilateral pulmonary infiltration	42 (42.4%)	182 (22.7%)	2.45	1.77–3.31	**<0.001**
Fever > 38°C	48 (48.5%)	437 (54.5%)	0.28	0.12–0.67	0.285
Increased ultrasensitive troponin	32 (32.3%)	215 (26.8%)	1.34	1.02–2.12	**0.04**
Multilobar pulmonary extension	24 (24.2%)	166 (20.7%)	1.54	1.11–2.18	**0.04**
PaO2/FiO2 < 300	71 (71.7%)	265 (33.1%)	3.79	2.42–4.91	**<0.001**
Inappropriate therapy	83 (83.8%)	19 (2.4%)	277	134.7–434.1	**<0.001**
Quinolones or macrolide or cephalosporins in the previous 30 days	71 (71.7%)	272 (33.9%)	5.14	3.34–8.81	**<0.001**
NIV	7 (7%)	20 (2.5%)	2.81	1.31–5.9	**0.022**
Platelets < 150.000 mm^3^	28 (28.3%)	130 (13.5%)	1.18	0.95–1.69	**0.001**
SOFA score	3.5	2.3	2.8	2.23–2.94	**<0.001**
Mean length of hospitalization (days)	18.9	16.7	2.95	1.82–2.67	**0.005**
Mean length of therapy (days)	19.2	14.2	1.62	1.43–1.99	**0.006**
ICU admission	12 (12.2%)	12 (1.5%)	1.89	1.54–3.22	**0.002**
Severe sepsis or septic shock	25 (25.2%)	74 (7.7%)	2.63	2.34–3.78	**<0.001**
30-day mortality	40 (40.4%)	89 (11.1%)	2.72	1.62–3.82	**<0.001**
In-hospital mortality	54 (54.5%)	102 (12.7%)	6.9	3.91–8.92	**<0.001**

Legend. MDR: multidrug-resistant; PSI: pneumonia severity index; COPD: chronic obstructive pulmonary disease; HCAP: healthcare-associated pneumonia; PPI: proton pump inhibitors; NIV: non-invasive ventilation; SOFA: sequential organ failure assessment; ICU: intensive care unit.

The univariate analysis between patients with non-MDR etiology versus those without etiologic diagnosis is presented in **S1 Table** (see supplementary material). Compared to MDR-group, almost all patients included in the MDR+ group belonged to the high severity classes (IV or V) of PSI, received more frequently a previous antibiotic therapy with quinolones, macrolides or 3^rd^ generation cephalosporins, had higher median Charlson score, were more likely to receive an inappropriate therapy, had higher mean duration of antibiotic therapy, higher mean LOS, and a significantly higher mortality rate.

Most patients included in the MDR+ group had an Aliberti score ≥3 (70.7% vs 42.2%, p<0.001), a Shorr score ≥1 (69.7% vs 37.8%, p<0.001), and a Shindo score ≥2 (60.6% vs 37.3%, p<0.001). As shown in **Table 3**, the final logistic regression model identified four variables significantly associated with MDR etiology including 1) one risk factor for HCAP, 2) bilateral pulmonary infiltration, 3) the presence of pleural effusion, and 4) the severity of respiratory impairment calculated by use of PaO2/FiO2 ratio.

**Table 3:** Multivariate analysis of factors associated with MDR isolation.

**Variables**	**p**	**OR**	**95% CI**	**VIF**
Bilateral Pulmonary Infiltration	0.018	1.75	1.09–2.79	1.05
Pleural effusion	<0.001	1.95	1.25–3.07	1.02
At least one of HCAP criteria: Previous hospitalization (3 months), dialysis, i.v. therapy previous 30 days, residence in nursing home or long-term care facility	<0.001	2.52	1.57–4.09	1.07
PaO2/FiO2 ratio < 300	<0.001	4.08	2.55–6.67	1.03

Legend. MDR: multidrug-resistant; HCAP: health-care associated pneumonia.

**Table 4**summarizes the risk score (ARUC score) for MDR pneumonia and the designation of points. The total possible score ranged from 0 to 3.5.

**Table 4:** ARUC score for early identification of patients with MDR-pneumonia.

**HCAP criteria (at least one of the following):** Previous hospitalization (3 months), dialysis, i.v. therapy previous 30 days, residence in nursing home or long-term care facility	**+ 1 pts**
**Bilateral Pulmonary Infiltration**	**+ 0.5 pts**
**Pleural effusion**	**+ 0.5 pts**
**PaO2/FiO2 < 300**	**+ 1.5 pts**

Legend. MDR: multidrug-resistant; HCAP: health-care associated pneumonia; COPD: chronic obstructive pulmonary disease.

**Fig 3:**
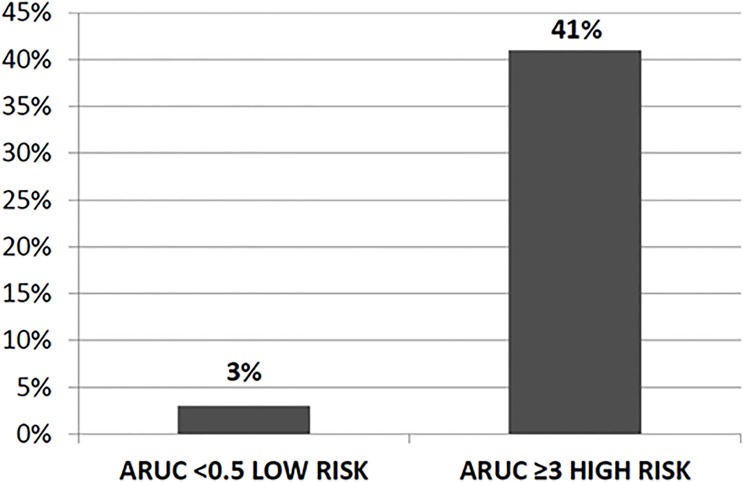
Risk stratification of MDR isolation on the basis of ARUC score. Legend. MDR: multidrug-resistant.

The prevalence of MDR pathogens rose with increasing score after stratifying the scores into Low (<0.5), and High (≥3) risk (see **Fig 3**).

When the score was <0.5 the prevalence of MDR pathogens was very low (3%), while the prevalence climbed to 41% when the score was ≥3.

**Fig 4:**
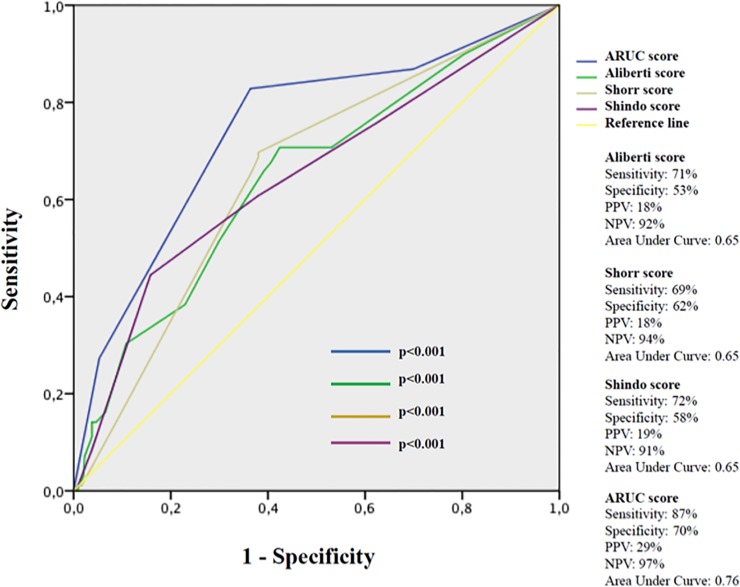
ROC curves of ARUC, Aliberti, Shorr and Shindo scores. Legend. PPV: positive predictive value; NPV: negative predictive value.

As a screening test, a negative ARUC score (<0.5) had a good sensitivity (75%) and a strong NPP (95%), while a positive score ≥3 had a high specificity (92%) and a strong PPV (93%). **Fig 4**compares sensitivity, specificity, PPV, and NPV of ARUC, Aliberti, Shorr, and Shindo scores.

The area under curve (AUC) of our model was higher (0.76, 95% Confidence Interval [CI] 0.71–0.82), than that of Aliberti (AUC = 0.65, 95%CI 0.59–0.71), Shorr (AUC = 0.65, 95%CI 0.60–0.70), and Shindo scores (AUC = 0.65, 95%CI 0.59–0.71). The goodness-of-fit Hosmer-Lemeshow χ2 was 7.64 (p = 0.469) for ARUC score, 7.45 (p = 0.489) for Shorr score, 10.25 (p = 0.248) for Aliberti score, and 11.91 (p = 0.155) for Shindo score.

**Fig 5:**
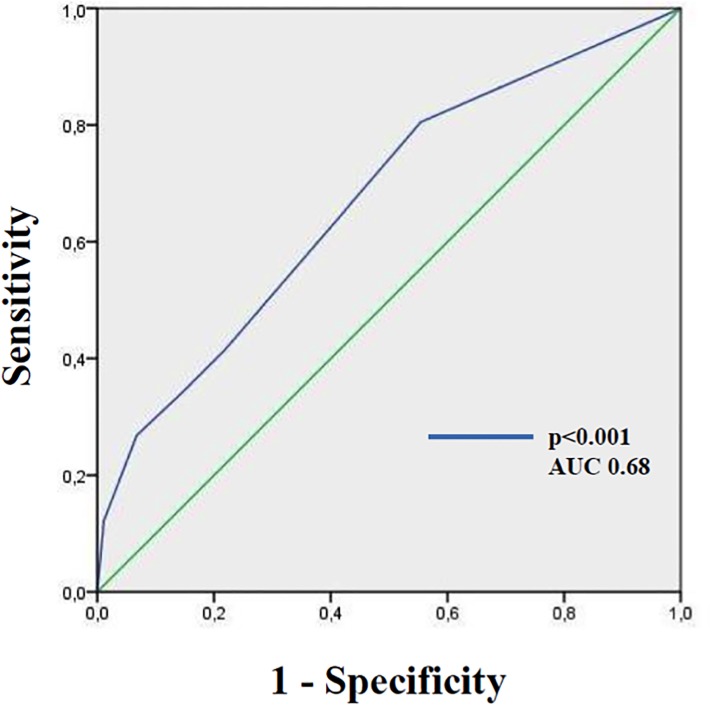
ROC curves of ARUC score on the Spanish population.

The performance of the score was evaluated on 929 patients with pneumonia with a median age of 80 (IQR 70–87) years. Most of patients had multiple comorbidities and 35% were classified as having HCAP. An attempt to microbiological diagnosis was made in 857 patients with an overall rate of etiological diagnosis of 23.2%. In 49 out of 199 patients (24.6%) with microbiological diagnosis a MDR pathogen was isolated (5.2% of the overall cohort). Clinical and microbiological characteristics of the validation cohort are listed in **S2 Table** (see supplementary material). The distribution of ARUC score’s variables in the validation cohort is detailed in **S3 Table** (see supplementary material). Overall, the 4% of population belonged to the low risk class of ARUC score, while the 39% belonged to the high risk class. A positive ARUC score (≥0.5) showed a sensitivity of 85% and a specificity of 50%, a PPV of 28% and a NPV of 94% to predict isolation of MDR pathogen. As reported in **Fig 5,** the AUC of this model was 0.68 (95% CI 0.60–0.77).

## Discussion

The relevant and novel findings of this study are that 1) in the context of patients with community-onset pneumonia exists a proportion of patients who develop an infection due to MDR pathogens, 2) MDR isolation is associated with inappropriate empirical antibiotic therapy and accounts for an unacceptable related mortality, 3) HCAP definition alone is not sufficient to discriminate patients at risk for MDR pathogens, and 4) the ARUC score, a new prediction rule, appears as a useful tool to help physicians in an early recognition of patients living in the community with pneumonia due to MDR pathogens.

The first key point of our study is that patients with community-onset pneumonia due to MDR pathogens have a high 30-day mortality rate (40.4%) and a high likelihood to receive an inappropriate initial antibiotic therapy (83.8%). Aliberti and coworkers, analyzing a population of 935 patients, observed that patients with risk factors for MDR had higher mortality rate than those who had not (22% vs 10%). Compared to abovementioned study, we found a higher proportion of MDR isolates (11%) and also higher mortality rates. The high mortality observed in our patients appears directly related to a high frequency of empirical inadequate antibiotic therapy. Moreover, another factor which may also explain the high mortality rate is the fact that most of our patients were old and critically ill and that we focused our analysis in patients who were hospitalized in medical wards for at least 48 hours; thus patients with mild or moderate pneumonia were excluded from the study.

Some reports describe the increasing prevalence of MDR organisms in subjects presenting *de novo* to the hospital [1,4,6,9,10,19,20,21]. The concept of HCAP, which was proposed in 2005 [16], was meant to serve as a tool to help clinicians to stratify individuals with high likelihood to have pneumonia due to a resistant pathogen, like MRSA, *P*. *aeruginosa* or other MDR gram-negative bacilli. Many studies have documented that HCAP is associated with increased mortality and higher risk of etiology due to MDR pathogens [1–4,6,8–10,19–22], and a prospective interventional study on elderly patients with pneumonia showed clinical benefit of an empirical broad-spectrum antibiotic therapy [23]. However, some authors have expressed concern that antibiotic treatment decisions driven by the concept of HCAP might lead to excessive prescription and abuse of broad-spectrum anti-infectives [5,7], leading to unnecessary costs and promote resistance. Our study confirms that HCAP per se has limited value in segregating subjects with potentially resistant infections: as a matter of fact a significant percentage of cases of pneumonia caused by MDR-bacteria (31.3%) were encountered in the CAP group [24, 25, 26].

Since HCAP definition alone is not sufficient to predict the risk for MDR pathogens, the development of risk stratification scoring tools provide to physicians an easy-to-use mechanism to determine which patients presenting with pneumonia may require broad-spectrum antibiotic coverage. We evaluated the performance of 3 existing scores and developed a new risk score (ARUC score) for the assessment of MDR pathogens in patients with community-onset pneumonia. Both C statistic of the model and goodness-of-fit Hosmer-Lemeshow χ2 statistic confirmed a good discriminatory power of ARUC score in the early identification of patients with pneumonia due to MDR organisms. Of importance a score <0.5 had a good sensitivity and a strong NPP, while a score ≥3 had a high specificity and strong PPV. These results highlight the role of ARUC score in selecting patients at lowest and highest risk for carrying MDR pathogens.

The strength of ARUC score may be that it is derived from a population of severely ill elderly patients, and in the majority of our patients were contemporary present at least two or more recognized risk factors for MDR; thus we had the chance to explore additional factors possibly related to an infection by a MDR strain. In addition ARUC score takes in account not only predisposing host factors for MDR but also clinical-radiological parameters evaluating the severity of illness, such as PaO2/FiO2 ratio, bilateral pulmonary infiltration and the presence of pleural effusion. A moderately good performance of this bedside score was confirmed in a multicenter Spanish population of elderly patients with pneumonia.

Another message of our article is that pneumonia due to MDR bacteria may be more severe than other causes, and is more frequently associated with higher degree of respiratory impairment expressed as PaO2/FiO2 ratio. This is not surprising since the involved species (*S*. *aureus*, *P*. *aeuginosa* and other MDR *Enterobacteriaceae*) are particularly virulent and frequently cause bilateral pneumonia; moreover, initial outpatient antibiotics usually prescribed in patients with CAP (e.g. macrolides, cephalosporins, or fluoroquinolones) are ineffective in patients with MDR etiology and this may lead to a more complicated clinical course. As matter of fact, Khawaja and coworkers found that the microbes causing severe CAP were different from the usual spectrum: *Staphylococcus aureus* and *Pseudomonas aeruginosa* were the common causative pathogens and were associated with high mortality [27].

Our study has important limitations: culture data were not always obtained in every case of suspected pneumonia, which introduces a selection bias. Thus percentage of MDR bacteria may be underestimated, and we cannot exclude that a proportion of patients who died without definite diagnosis had a MDR etiology. Another limitation is that the ARUC score was derived by comparing the MDR population to the composite group of patients without MDR etiology or with unknown etiology; however, we did not find significant differences between these latter groups in term of baseline characteristics and outcomes, and this finding reduces the potential bias of this analysis. Finally, since ARUC score showed a good but not optimal discrimination power (AUC 0.68 in the validation cohort) future large studies are needed to assess the better scoring tool for the early identification of patients with pneumonia due to MDR pathogens.

In conclusion a rapid identification of patients with infection due to MDR pathogens seems crucial to reduce mortality for community-onset pneumonia. Physicians working in ED should adopt simple risk scores, like ARUC score, to select the most appropriate antibiotic regimens. This individualized approach may help clinicians to identify those patients who need an empirical broad-spectrum antibiotic therapy [28].

## Supporting Information

S1 Table Univariate analysis of MDR-group compared to patients without etiology. PSI: pneumonia severity index; MRSA: methicillin-resistant *Staphylococcus aureus*.
(DOCX)

S2 Table Characteristics of the validation cohort. PSI: pneumonia severity index; MRSA: methicillin-resistant Staphylococcus aureus.
(DOC)

S3 Table Distribution of the ARUC score’s variables in the Spanish population. HCAP: healthcare-associated pneumonia; COPD: chronic obstructive pneumonia disease.
(DOC)
