# Supplementary material for: Individualizing Risk of Multidrug-Resistant Pathogens in Community-Onset Pneumonia
Source: PLoS One. 2015 Apr 10;10(4):e0119528. doi: 10.1371/journal.pone.0119528 (PMC4393134; doi:10.1371/journal.pone.0119528)
Supplement: S1 Table — PSI: pneumonia severity index; MRSA: methicillin-resistant Staphylococcus aureus. (DOCX) [file pone.0119528.s001.docx]

**S1 Table.** Univariate analysis of MDR- group compared to patients without etiology

| **Variables** | **MDR-**  **n=201 patients** | **No etiology**  **n=600 patients** | **p** |
| --- | --- | --- | --- |
| Age (median) | 74.9 | 75 | 0.4 |
| Male sex | 127 (63.2%) | 363 (60.5%) | 0.1 |
| PSI IV-V class | 170 (84.5%) | 467 (77.8%) | 0.07 |
| Adherence to guidelines | 105 (52.2%) | 316 (52.6%) | 0.8 |
| ≥ 2 comorbidities | 41 (20.4%) | 120 (20%) | 0.8 |
| Aliberti score ≥ 3 | 76 (37.8%) | 262 (43.6%) | 0.07 |
| Shorr score ≥ 1 | 66 (32.8%) | 237 (39.5%) | 0.06 |
| Shindo score ≥ 2 | 73 (36.3%) | 226 (37.6%) | 0.7 |
| Heart failure | 45 (22.4%) | 172 (28.6%) | 0.06 |
| Chronic hepatitis | 9 (4.5%) | 58 (9.6%) | 0.08 |
| Diabetes | 40 (19.9%) | 111 (18.5%) | 0.4 |
| Renal failure | 33 (16.4%) | 111 (18.5%) | 0.1 |
| COPD | 70 (34.8%) | 185 (30.9%) | 0.06 |
| Dementia | 38 (18.9%) | 142 (23.6%) | 0.06 |
| HCAP | 76 (37.8%) | 220 (36.6%) | 0.3 |
| Neoplasm | 42 (20.9%) | 128 (21.3%) | 0.8 |
| Pleural effusion | 84 (41.8%) | 245 (40.8%) | 0.6 |
| Malnutrition | 26 (12.9%) | 75 (12.5%) | 0.9 |
| PPI/H2 blockers | 66 (32.8%) | 173 (28.9%) | 0.09 |
| Previous surgery (30 days) | 3 (1.4%) | 16 (2.6%) | 0.08 |
| Bilateral pulmonary infiltration | 47 (23.4%) | 135 (22.5%) | 0.9 |
| Fever > 38°C | 117 (58.2%) | 320 (53.4%) | 0.06 |
| Increased ultrasensitive troponin | 52 (25.9%) | 163 (27.2%) | 0.3 |
| Multilobar pulmonary extension | 36 (17.9%) | 130 (21.6%) | 0.09 |
| PaO2/FiO2 < 300 | 70 (34.8%) | 195 (32.5%) | 0.2 |
| Quinolones or macrolide or cephalosporins in the previous 30 days | 70 (34.8%) | 202 (33.6%) | 0.7 |
| SOFA score | 2.3 | 2.4 | 0.8 |
| Mean length of hospitalization (days) | 15.6 | 16.1 | 0.4 |
| Mean length of therapy (days) | 13.9 | 14.3 | 0.1 |
| ICU admission | 3 (1.5%) | 9 (1.5%) | 1.0 |
| Severe sepsis or septic shock | 22 (10.9%) | 52 (8.6%) | 0.08 |
| 30-day mortality | 23 (11.4%) | 66 (11%) | 0.8 |
| In-hospital mortality | 30 (14.9%) | 72 (12%) | 0.08 |

**Legend.** MDR: multidrug-resistant; PSI: pneumonia severity index; HCAP: healthcare-associated pneumonia; COPD: chronic obstructive pulmonary disease; PPI: proton pump inhibitors; SOFA: sequential organ failure assessment; ICU: intensive care unit.
