# Supplementary material for: Individualizing Risk of Multidrug-Resistant Pathogens in Community-Onset Pneumonia
Source: PLoS One. 2015 Apr 10;10(4):e0119528. doi: 10.1371/journal.pone.0119528 (PMC4393134; doi:10.1371/journal.pone.0119528)
Supplement: S2 Table — PSI: pneumonia severity index; MRSA: methicillin-resistant Staphylococcus aureus. (DOC) [file pone.0119528.s002.doc]

**S2 Table.** Characteristics of the validation cohort.

|  | **ENEMI II**  **n=929 (%)** |
| --- | --- |
| Age (median, IQR) | 80,70-87 |
| Male | 519 (55.9) |
| McCabe Jakson  Rapidly fatal  Ultimately fatal  No fatal | 65 (7)  383 (41.2)  476 (51.2) |
| Charlson's index | 6, 4-8 |
| Barthel index | 80, 20-100 |
|  | **N=857 (%)** |
| PSI |  |
| Low risk (Class I-III) | 136 (19) |
| High risk (Class IV-V) | 721 (81) |
| CURB-65 |  |
| Low risk (Class 0-1) | 238 (27.7) |
| Mild risk (Class 2) | 338 (36.3) |
| High risk (Class 3-5) | 281 (32.7) |
|  | **N=199 (%)** |
| *S. pneumoniae* | 103 (51.8) |
| *P. aeruginosa* | 24 (12.1) |
| *Enterobacteriaceae* | 18 (9) |
| *H. influenzae* | 11 (5.5) |
| MRSA | 9 (4.5) |
| *Legionella spp.* | 8 (1.4) |
| Influenza virus | 12 (6) |

**Legend.** PSI: pneumonia severity index; MRSA: methicillin-resistant *Staphylococcus aureus*.
