# Supplementary material for: Individualizing Risk of Multidrug-Resistant Pathogens in Community-Onset Pneumonia
Source: PLoS One. 2015 Apr 10;10(4):e0119528. doi: 10.1371/journal.pone.0119528 (PMC4393134; doi:10.1371/journal.pone.0119528)
Supplement: S3 Table — HCAP: healthcare-associated pneumonia; COPD: chronic obstructive pneumonia disease. (DOC) [file pone.0119528.s003.doc]

**S3 Table. Distribution of the ARUC score’s variables in the Spanish population**

| **Variables** | **Points** | **N=929 (%)** |
| --- | --- | --- |
| Bilateral Pulmonary Infiltration | 0.5 | 288 (31) |
| Pleural effusion | 0.5 | 188 (21.6) |
| HCAP criteria | 1 | 327 (35.1) |
| PaO2/FiO2 < 300 | 1.5 | 285 (30.7) |

***Some patients presented ≥1 condition**

**Legend**. HCAP: healthcare-associated pneumonia; COPD: chronic obstructive pneumonia disease.
